# Fatigue in primary sclerosing cholangitis is associated with sympathetic over‐activity and increased cardiac output

**DOI:** 10.1111/liv.12709

**Published:** 2014-12-04

**Authors:** Jessica K. Dyson, Ahmed M. Elsharkawy, Christopher A. Lamb, Ahmad Al‐Rifai, Julia L. Newton, David E. Jones, Mark Hudson

**Affiliations:** ^1^ The Liver UnitFreeman HospitalNewcastle Upon TyneUK; ^2^ The Liver UnitUniversity Hospitals BirminghamBirminghamUK; ^3^ Institute for Cellular MedicineNewcastle UniversityNewcastle upon TyneUK; ^4^ Salford Royal NHS Foundation TrustManchesterUK; ^5^ UK NIHR Biomedical Research Centre in AgeingNewcastle‐upon‐TyneUK

**Keywords:** autonomic dysfunction, fatigue, primary sclerosing cholangitis, quality of life

## Abstract

**Background & Aims:**

Patients with primary sclerosing cholangitis (PSC) frequently highlight the impact of fatigue on their life quality. The study aims were to evaluate fatigue and its associations in PSC and investigate whether overt autonomic dysfunction contributes to the expression of fatigue.

**Methods:**

All PSC patients under active follow‐up at a regional liver centre were sent disease‐ and symptom‐assessment tools. Three control groups were utilized; unselected community controls, patients with inflammatory bowel disease (IBD) without PSC, and cholestatic controls with primary biliary cirrhosis (PBC). A representative subgroup of PSC patients and normal controls underwent formal autonomic assessment.

**Results:**

Symptom‐assessment tools were returned by 40 non‐transplanted patients. PSC patients had significantly worse fatigue than population controls (*P* = 0.005). Fatigue was significant compared to population controls whether or not patients had accompanying IBD, although was more marked in those with both PSC and IBD. In patients with PSC and IBD, fatigue severity and autonomic symptoms were significantly increased in those with prior significant surgical intervention. Clinically significant autonomic dysfunction was seen in 22.5% of PSC patients, and of those, 78% had significant fatigue. Neurally mediated hypotension was found in 60% of PSC patients compared to 8% in the control group. The PSC group had increased sympathetic activity and reduced parasympathetic activity.

**Conclusion:**

Fatigue is a significant problem in a minority of PSC patients and appears to be associated with autonomic dysfunction. Fatigued PSC patients should be screened for autonomic dysfunction and targeting such dysfunction represents a potential approach to treatment which warrants further exploration.

## Abstract 2

See Editorial on Page 1489

**Abbreviations**
BP: blood pressure
COMPASS: Composite Autonomic Symptom Scale
ERCP: endoscopic retrograde cholangiopancreatography
ESS: Epworth Sleepiness Scale
FIS: Fatigue Impact Scale
HADS: Hospital Anxiety and Depression Scale
HR: heart rate
IBD: inflammatory bowel disease
LFTs: liver function tests
MRCP: magnetic resonance cholangiopancreatography
NAFLD: non‐alcoholic fatty liver disease
PBC: primary biliary cirrhosis
PSC: primary sclerosing cholangitis
ULN: upper limit of normal

**Key Points**

- Patients with primary sclerosing cholangitis (PSC) have significantly worse fatigue than community controls which impacts on their life quality.
- The level of fatigue experienced correlates directly with the autonomic dysfunction symptom severity.
- Dynamic autonomic testing suggested a shift in the balance of autonomic reactivity from parasympathetic (down‐regulated) to sympathetic (up‐regulated).
- At risk patients can be identified using autonomic dysfunction screening tools which are very acceptable to patients and can be used in the ordinary clinic setting. Well‐described orthostatic intolerance treatment paradigms may offer possible approaches to therapy.

Primary sclerosing cholangitis (PSC) is a chronic cholestatic condition predominantly affecting males 1. The underlying pathological process is inflammation and progressive fibrosis, with stricturing of both intrahepatic and extrahepatic bile ducts causing chronic cholestasis, eventually resulting in cirrhosis. There are currently no treatments effective in slowing progression of PSC to end‐stage disease, the only treatment for which is transplantation. Cholangiocarcinoma development is another significant complication of PSC, as is the development of colonic carcinoma, often in the context of associated inflammatory bowel disease (IBD) 2. The aetiology of PSC is uncertain, as are the relative contributions of genetic and environmental factors to pathogenesis 3.

While the principal clinical focus in PSC is, rightly, on the risks of disease progression and associated cancers, patients themselves frequently highlight the impact of systemic symptoms such as fatigue on their life quality 4. Patient reports in PSC mirror, in many regards, those in primary biliary cirrhosis (PBC), another chronic cholestatic condition in which fatigue is an important and well‐characterised problem 5, 6. The actual impact of fatigue in population terms in PSC is, however, unclear with only a limited number of studies undertaken with often contrasting findings regarding fatigue severity and accompanying life quality impact 7, 8, 9, 10, and the response to specific therapies 11. Furthermore, it is currently unclear as to what processes might underlie fatigue in PSC. In the case of both PBC and other chronic liver diseases these include sleep disturbance [in particular daytime somnolence 12, 13] and autonomic nervous system dysfunction, relating in particular to vasomotor control 14, 15, 16, 17, 18. In PSC, there is also the potential for associated clinical conditions such as IBD and anaemia, which are themselves associated with fatigue, to contribute to overall symptom burden.

The aim of this study was to evaluate fatigue and its associations in a comprehensive cohort of PSC patients. Furthermore, we investigated autonomic and cardiovascular responses in PSC subjects to explore whether autonomic dysfunction is a feature of PSC and whether this is associated with fatigue severity as is the case in PBC and non‐alcoholic fatty liver disease (NAFLD) 14.

## Patients and methods

### Subjects

Patients with PSC currently under active follow‐up at a regional liver centre were identified using a comprehensive case‐finding approach. Patients under follow‐up following liver transplantation for PSC were also identified and are reported separately. All participants provided written informed consent. This study was approved by the Newcastle and North Tyneside Local Research Ethics Committees (approval 05/Q0906/205, 1 June 2006). The diagnosis of PSC was made on the basis of characteristic ERCP or MRCP findings and compatible liver histology (where available), along with consistent abnormalities in liver function tests (LFTs), with all patients having an alkaline phosphatase (ALP) of >1.5× ULN at diagnosis. All PSC patients were investigated by colonoscopy at diagnosis for the presence of inflammatory bowel disease (IBD), with biopsies undertaken as clinically indicated at the time of the investigation. The presence of microscopic IBD led to patients being categorized as having IBD for the purposes this study groups. In addition, patients with colonic symptoms developing during follow‐up were also investigated with colonoscopy. Most recent blood biochemistry and haematology were retrieved and reviewed. Three control groups were utilized. Members of the local population were invited to participate and recruited via notices in the press. These were community controls as they were not screened positively or negatively for intercurrent diseases and/or relevant therapy. Patients with IBD, all of whom had extensive colitis (defined for the purposes of this study as extending beyond proctitis) that was either quiescent or mildly active (defined histologically), had had PSC actively excluded and had normal liver biochemistry and bile duct imaging on MRCP, were recruited from the specialist clinic of the investigators. The IBD patients were age‐ and sex‐matched to the PSC patients with IBD at a group level. The third group was age‐ and sex‐matched PBC patients from the local PBC clinic.

### Symptom‐assessment tools

All symptom‐assessment tools have been validated for self‐completion and used previously in patients with liver disease.

#### Fatigue Impact Scale

The Fatigue Impact Scale (FIS) is a 40‐item generic fatigue impact scale that was used to assess fatigue severity in the PSC and control groups. The FIS has previously been extensively utilized in both normal controls and PSC populations 19.

#### Epworth Sleepiness Scale

Daytime somnolence was assessed using the Epworth Sleepiness Scale (ESS, possible score range 0–24) 20. A score of 10 or more in this fully validated scale is indicative of significant daytime hyper‐somnolence. A score of ≥5 and <10 was regarded as being indicative of moderate daytime hyper‐somnolence.

#### Hospital Anxiety and Depression Scale

The Hospital Anxiety and Depression Scale (HADS) is a 14‐item measure of current anxiety (HADS‐A) and depression (HADS‐D). The HADS was specifically developed for use in physical illness by excluding items related to somatic symptoms. In this study, a HADS score >10 was used for definition of case‐ness.

#### Autonomic Dysfunction Symptoms (COMPASS)

The frequency and severity of symptoms of autonomic dysfunction were assessed using the Composite Autonomic Symptom Scale (COMPASS) 21. The COMPASS consists of 73 questions grouped into domains relating to individual aspects of the autonomic nervous system weighted according to clinical relevance 21. In all domains, higher scores indicate a greater symptom load. COMPASS has been fully validated against laboratory‐based haemodynamic autonomic function tests and has been used to investigate autonomic dysfunction in a variety of diseases 22.

### Objective assessment of autonomic parameters

Ten non‐transplanted PSC patients, selected to be representative of the PSC population as a whole using a matrix approach, and age‐ and sex‐matched controls underwent formal autonomic assessment with investigation for neurally mediated hypotension using our previously described protocol 15, 23. All investigations were performed at the same time of day, and took place in a warm, quiet room. All cardiovascular assessments were carried out with continuous heart rate (HR) and beat‐to‐beat blood pressure (BP) measurement (Taskforce, CN Systems, AHG Health, Somerset West, South Africa). Orthostatic hypotension (fall of >20 mmHg systolic or 10 mmHg diastolic on orthostasis) and vasovagal syncope (loss of consciousness resulting from bradycardia induced during the tilt) were diagnosed using recognized diagnostic criteria 15, 23.

### Data analysis

All data were normally distributed (D'Agostino & Pearson) and are presented as mean and standard deviation. Data were analysed using Graphpad software (Prism, GraphPad Software Inc., La Jolla, CA, USA). Comparisons between individual patient groups were by student's *t*‐tests, and proportions of groups by chi‐squared test. Correlations between variables were by Spearman Rank test. A statistically significant result was taken when *P* < 0.05.

## Results

All patients with a confirmed diagnosis of PSC and still under follow‐up between 1998 and 2012 were identified (*n* = 81). Fully completed symptom‐assessment tools were returned by 51 patients. Eleven of these patients had undergone liver transplantation and were assessed separately. Median follow‐up was 5.23 years (range 0–19 years). **Table** 1 shows the demographic details and distribution of subtypes of cholangiopathy of the non‐transplanted PSC study group (*n* = 40).

**Table 1:** Demographic details and distribution of subtypes of cholangiopathy in the non‐transplanted PSC study group (*n* = 40). Figures in brackets for blood parameters refer to the standard deviation

Demographic details	
Males (%)	31 (78%)
Mean age (SD) years	51 (13)
Concurrent inflammatory bowel disease (IBD) (%)	24 (60%)
Blood parameters	
Albumin (g/L)	41 (7)
Alkaline Phosphatase (U/L)	275 (248)
Bilirubin (micromol/L)	31 (53)
Alanine Aminotransferase (U/L)	62 (49)
Random Glucose (mmol/L)	6.1 (2.6)
Platelets (×103/mm^3^)	242 (138)
Immunoglobulin G (IgG)	15 (4.1)
Follow‐up years	5.2 (0–19)
Number of medications	4.5 (3.6)
Distribution of subtypes of cholangiopathy	
Small duct PSC	11 (28%)
Large duct PSC	
Total	27 (68%)
Extrahepatic disease	1 (3%)
Intrahepatic disease	9 (23%)
Both	17 (43%)
Data unavailable	2 (5%)

### Symptoms and their associations in PSC

**Figure 1:**
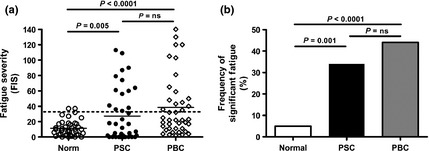
(a) Comparison of fatigue severity (assessed using FIS) between patients with PSC (*n* = 40), community (*n* = 40) and PBC control populations (*n* = 40). Broken line represents the mean + 2SD for fatigue severity in the control population, this study definition for significant fatigue. (b) Frequency of significant fatigue defined using this study cut‐off in PSC patients and community and PBC controls.

**Figure 2:**
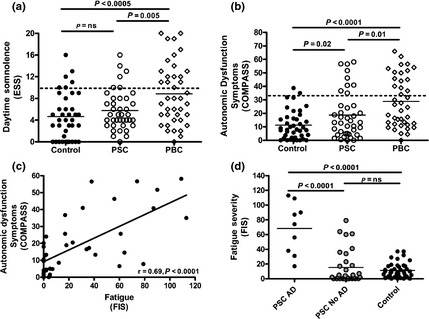
(a) Severity of daytime somnolence (assessed using the ESS) in PSC patients (*n* = 40) and community (*n* = 40) and PBC controls (*n* = 40). (b) Severity of autonomic symptoms (assessed using COMPASS) in PSC patients and community and PBC controls and (c) their relationship with fatigue severity in PSC patients. (d) Fatigue severity scores in PSC patients with and without autonomic dysfunction (AD) symptoms.

The non‐transplanted PSC group had significantly worse fatigue scores than the community controls (*n* = 40; Fig. 1a). Fatigue levels were lower than in age and sex‐matched PBC patients (*n* = 40), although the difference was not significant. Using an age‐ and gender‐relevant cut‐off for significant fatigue based on the control population, 35% of the PSC patients were found to be fatigued (Fig. 1b). The demographic distribution for fatigued PSC patients mirrored the patient group as a whole. In PSC patients, unlike PBC controls, daytime somnolence scores were not significantly elevated (Fig. 2a). In contrast, significant autonomic dysfunction symptoms were seen (Fig. 2b), with nine PSC patients (22.5%) exceeding the previously defined cut‐off for clinically significant autonomic dysfunction in the context of chronic fatigue 24. Moreover, the level of fatigue experienced by PSC patients correlated directly with the autonomic dysfunction symptom severity (Fig. 2c). Seven of those nine (78%) PSC patients with significant autonomic dysfunction symptoms had significant fatigue, compared with only 7 of 31 (22.5%) of the group without significant autonomic dysfunction (*P* < 0.005; Fig. 2d). Six patients were taking antihypertensive medication. No differences in either autonomic dysfunction or fatigue severity scores was seen between those taking and those not taking antihypertensive medication. Depression was not a frequent cause of fatigue in PSC with only two patients (one fatigued and one non‐fatigued) reaching caseness. No association was seen between serum biochemistry parameters and fatigue severity in either the PSC or the PBC group. In the PSC group, fatigue severity was unrelated to whether disease was intra‐ or extrahepatic in type (FIS 21.1 ± 9.9, 27% fatigued; 28.7 ± 6.8, 37% fatigued respectively, both *P* = ns).

**Figure 3:**
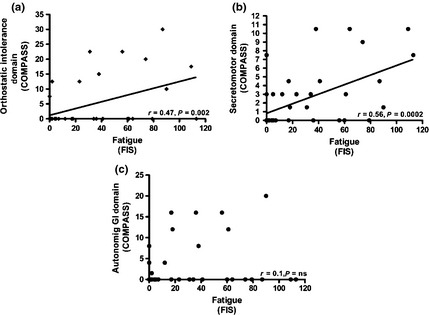
Fatigue severity in PSC (*n* = 40) correlates directly with the (a) *Orthostatic Intolerance* and (b) *Secretomotor* domains of the COMPASS but not other domains including the (c) *Autonomic GI*.

The association between autonomic dysfunction symptoms and fatigue was not seen in all COMPASS domains, being restricted to *Orthostatic Intolerance* and *Secretomotor* (Fig. 3a, b). No association was seen with other domains such as *Autonomic GI* (Fig. 3c). Given its apparent importance in fatigue pathogenesis in PSC, we went on to explore the organic basis of the orthostatic intolerance symptoms using dynamic testing.

### Dynamic testing of haemodynamic responses to standing in non‐transplanted PSC patients

**Figure 4:**
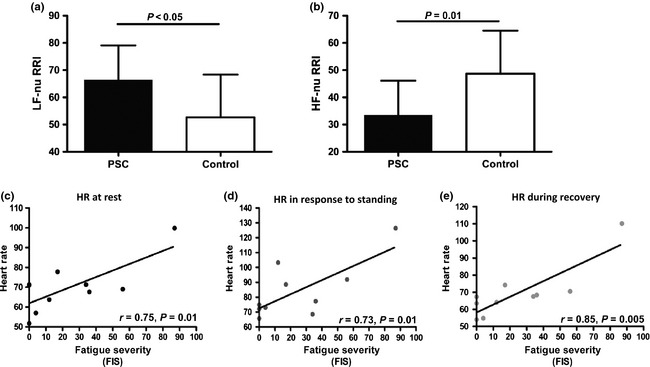
Objective assessment of autonomic dysfunction in a representative subgroup of PSC patients (*n* = 10). Compared to age‐ and sex‐matched community controls (*n* = 10) PSC patients have (a) significantly increased low frequency heart rate variability and (b) significantly decreased high frequency heart rate variability. Correlation between heart rate and fatigue severity in PSC patients (c) at rest (d) after 40 min tilt and (e) during recovery.

Ten representative non‐transplanted PSC patients (mean FIS 24.6 ± 29.0) and matched normal controls underwent formal autonomic assessment. Neurally mediated hypotension was found in six PSC patients (60%) compared to 1 (8%) in the control group (*P* = 0.05). Assessment of autonomic parameters at baseline (over 10 min resting supine) confirmed that, compared to controls population, the PSC group had increased sympathetic activity [increased low frequency (LF) heart rate variability] and reduced parasympathetic activity [reduced high frequency (HF) heart rate variability; Fig. 4a, b]. Heart rate at rest, in response to standing for 40 min and during recovery correlated significantly with increasing fatigue in the PSC group (Fig. 4c–e). Taken together, the dynamic autonomic testing confirmed the presence of overt autonomic vasomotor control abnormality, linked to fatigue severity, and suggested a shift in the balance of autonomic reactivity from parasympathetic (down‐regulated) to sympathetic (up‐regulated).

### Symptom impact in PSC patient subgroups

Primary sclerosing cholangitis is not a condition which exists in isolation. The association with IBD, a condition itself associated with both fatigue and autonomic dysfunction, presents a potential confounding process. Significant fatigue was seen in both patients without and with intercurrent IBD (Fig. S1a,b). Fatigue was more severe and more frequently seen in patients with IBD compared with patients without, although these differences did not reach statistical significance. The level of fatigue seen in PSC patients with IBD mirrored that seen in the control group of patients with IBD in the absence of PSC, (*n* = 20) who were group‐matched in terms of age (53.4 ± 16.7 vs 52.5 ± 14.2) and gender (75% male in each group) to the PSC patients with IBD, and all of whom had quiescent or only mildly active colitis. Both PSC and IBD and PSC alone patients exhibited significant autonomic symptoms (Fig. S1c). In both groups, the severity of fatigue correlated with the level of autonomic symptoms (Fig. S1d). Within the group of patients with both PSC and IBD, the severity of both fatigue and autonomic symptoms was significantly increased in the subgroup of patients who had undergone significant surgical intervention (colectomy and ileostomy/pouch formation) (Fig. S2a,b). Within the PSC group, no difference in fatigue severity was seen between patients who were and were not receiving UDCA therapy (Fig. S2c). This study was not designed or powered to address the question of fatigue in post‐transplant PSC patients but it was striking that fatigue was a significant ongoing problem in post‐transplant patients, with severity levels that were higher than those seen in the non‐transplanted PSC patients, although this difference did not reach statistical significance (Fig. S2d).

## Discussion

In this study, we set out to explore the prevalence, degree and associations of fatigue in a comprehensive and representative single‐centre cohort of PSC patients and, in particular, to explore potential factors contributing to fatigue in patients experiencing it. The patients in this study were not selected for clinical phenotype and were not subject to referral bias, all coming from the local centre catchment area. The assessment tools used are well‐described and validated measures optimized for patient self‐completion. This study was controlled with age‐ and sex‐matched community controls from the same geographical area, as well as patients with IBD in whom PSC had been actively excluded and patients with PBC. Within this cohort, fatigue levels were significantly higher than in the community controls, and similar to those in the PBC patients, with 35% of PSC patients experiencing significant fatigue (defined using a cut‐off of mean + 2SD for the community controls). Fatigue is a problem in PSC and one which impacts on a significant minority of patients. It is unrelated in severity to conventional markers of disease severity and disease distribution. The key association of fatigue in PSC appears to be with autonomic dysfunction suggesting a potential target for therapeutic intervention.

Unlike PBC, where fatigue is strongly associated with both autonomic dysfunction and daytime somnolence (12, 15, 16, 25, 26,) fatigue in the study cohort was only associated with autonomic dysfunction. Among the significant minority of PSC patients with significant autonomic symptoms, fatigue is very marked and almost universal (approximately 80%). In contrast, among patients without autonomic dysfunction, mean fatigue scores were no different to those seen in community controls. Overt depression was also an uncommon cause of fatigue in our cohort in contrast to previous findings 7. In our cohort, fatigue was slightly more severe in patients with both PSC and IBD (reaching levels similar to those seen in IBD only controls), but was, crucially, still significant in patients with PSC alone. The specific association with autonomic dysfunction was seen in both PSC and IBD and PSC alone patient groups. Although this study was not designed to explore the impact of UDCA use on fatigue (the specific reasons for UDCA use in individual patients, which may have included worse symptoms, were not recorded for example) there was no evidence that UDCA use was associated with lower fatigue levels. This mirrors the situation in PBC, where there is no evidence to suggest that UDCA reduces fatigue severity 27. Within the PSC and IBD patient group, the nature of the IBD and, in particular the need for previous surgery impacted significantly on fatigue. Patients with quiescent colitis who had not undergone surgical intervention had significantly lower levels of fatigue than post‐colectomy patients. Again, the higher levels of fatigue in the postsurgical patients were strongly related to increased levels of autonomic dysfunction symptoms in this group. The findings of this element from this study would suggest that the fatigue experienced by PSC patients has components related to PSC itself, as well as aspects relating to the presence of co‐existent IBD in some patients. It is likely that approaches to effective therapy will be equally complex and must take account of the different predisposing disease processes.

Although autonomic dysfunction symptoms were strongly associated with fatigue where present, in PSC, the effect was not uniform across the spectrum of such symptoms. In fact, the fatigue association was specific for the orthostatic intolerance and secretomotor domains of the COMPASS. The orthostatic intolerance association, which again mirrors that seen in PBC, other fatigue‐associated liver diseases such as NAFLD and non‐liver conditions such as chronic fatigue syndrome, suggests the potential presence of abnormal posture‐associated blood pressure regulation which, in the case of PBC, reflects acquired baroreceptor reflex insensitivity 23. A specific association with orthostatic intolerance would potentially explain the worsening of fatigue in PSC patients with IBD treated by colectomy where fluid homeostasis issues, which could exacerbate the expression of baroreceptor reflex insensitivity, are frequent.

Given the apparent strength of the link between orthostatic intolerance, autonomic symptoms and fatigue in PSC, we set out to undertake, for the first time in this disease, formal assessment of autonomic haemodynamic regulation using established approaches adopted in other disease settings including PBC. These studies confirmed the presence of significant heart rate variability abnormality in PSC (a feature of autonomic dysfunction) with features suggestive of increased sympathetic and decreased parasympathetic tone. Tilt‐testing, undertaken here in PSC for the first time, showed a significant correlation between heart rate at all phases of the tilt and fatigue severity, with tachycardia (again reflecting the parasympathetic to sympathetic tone shift) strongly associating with fatigue. These findings would all be in keeping with variable baroreceptor reflex insensitivity in PSC, with over‐utilization of sympathetic escape pathways, with levels of insensitivity and escape directly linked to fatigue. This model would explain the association between fatigue in PSC and both orthostatic intolerance symptoms (a direct manifestation of the baroreceptor reflex insensitivity) and secretomotor symptoms (a manifestation of sympathetic escape).

This study has limitations. The first is that the association between autonomic dysfunction and fatigue in PSC is currently correlative rather than mechanistic in nature. However, the apparent association between worse fatigue and increased autonomic dysfunction in patients with surgically treated IBD (colectomy and ileostomy or pouch procedure), where fluid‐balance challenges are well recognized, hints at a mechanism. Any causal role for autonomic dysfunction in fatigue in PSC will only be confirmed by undertaking trials of the efficacy of treatments able to reverse vasomotor autonomic dysfunction in terms of reducing fatigue severity. We believe that such trials are warranted. A further limitation was that the assessment of fatigue was only undertaken through subjective assessment approaches with no objective assessment of physical activity attempted. However, studies in PBC have demonstrated that perceived fatigue is associated with reduced physical activity levels. This raises the possibility that deconditioning may contribute to the clinical expression of fatigue and autonomic dysfunction in PSC, potentially exacerbating the phenotype. Again, intervention studies will be needed to dissect out these complex relationships. A further limitation is study size, a potential factor in explaining the differences in fatigue severity between this and other studies in the field. Very large scale cohort studies akin to those being performed in PBC on national scale cohorts would be very helpful in definitively addressing this important issue.

Our finding of significant levels of fatigue in PSC is seemingly at odds with the other large series in this disease 7, 10. There are several potential explanations for the apparent discrepancy including study size, case mix differences and psycho‐social functioning differences between populations [the same group reported no excess fatigue in PBC in their Scandinavian population; a finding at odds with most other studies in Northern European populations 28] and the nature of control groups. In practice, however, the studies have a similar finding of significant fatigue as a feature in a minority of PSC patients, with lower levels experienced at lower frequency than is the case in PBC 6, 28 and, as might be expected, cases of significant fatigue being seen in the community controls population. This study adds significantly to our understanding by identifying the subgroup of patients with PSC, who experience significant fatigue as those patients experiencing significant autonomic dysfunction. The strength of the association reported here between autonomic dysfunction and fatigue in PSC will help the clinical management of PSC patients through identifying patients at risk (autonomic dysfunction screening tools are very acceptable to patients and can be used in the ordinary clinic setting) and suggesting possible approaches to therapy through application of well‐described orthostatic intolerance treatment paradigms.

The findings of this study, and in particular the similarities and differences between fatigue associations in PSC and those previously reported in PBC, add to our understanding of the complex systemic symptoms of chronic cholestatic disease. Autonomic dysfunction is frequent in PBC and is strongly associated with both fatigue and low level cognitive impairment 29. It is associated with both organic brain lesions on magnetic resonance imaging and impairment of cerebral autoregulation 29, 30. These findings are compatible with structural change in brain areas regulating autonomic function as a consequence either of cholestasis or the underpinning disease process in PBC. This organic brain injury model would potentially explain the ongoing presence of fatigue in PBC following liver transplantation 31, and the direct link between the severity of that fatigue and degree of ongoing autonomic dysfunction. The findings of this study, showing similar effects in a second cholestatic liver disease, would support cholestasis rather than a disease‐specific aetiological factor in central autonomic dysfunction in PBC. The apparent continuation of PSC fatigue following liver transplantation (within the limitations of this study design), and the ongoing association with autonomic symptoms in the post‐transplant patients would further support the cholestatic injury model for autonomic dysfunction.

Fatigue levels seen in this study in PSC are somewhat lower than those in the PBC control groups (which themselves mirrored previous findings in PBC made in studies using similar methodologies). An explanation for this could be the absence in PSC patients of the additional processes thought to contribute to fatigue expression. These include daytime somnolence which was not a significant factor in PSC patients, and an apparent direct metabolic effect of antimitochondrial antibodies which are absent in PSC patients 32. In our model for metabolic fatigue in PBC, an underlying metabolic abnormality linked to the specific immune response in the condition is compensated for by vascular and transporter processes which are subject to autonomic regulation. Severe fatigue, according to this model, results from a dual process of primary metabolic fatigue exacerbated by a failure to compensate as a consequence of autonomic dysfunction occurring as a second disease process. In this model, autonomic dysfunction occurring in any disease setting could, if severe enough, impair the recovery from normal muscle metabolic activity in the context of exercise (as opposed to the enhanced metabolic state seen in PBC). We propose this as a generic model for autonomic dysfunction‐associated fatigue. If this model is correct, the conclusion of this study would be that PSC patients do indeed experience the same type of fatigue as is seen sporadically in the normal population, but they do so more frequently and to a greater degree because of their predisposition to cholestasis‐induced autonomic dysfunction exacerbated in some patients by the fluid‐balance effects of their IBD surgery.

In conclusion, fatigue is a significant problem in a minority of PSC patients. Symptom association data, together with the pilot objective autonomic function assessments, suggest it is associated with autonomic dysfunction. Clinical association data suggest it is exacerbated by other interventions and disease states, which make autonomic dysfunction or its features worse. Further confirmatory studies in larger cohorts are required. If the associations outlined here are confirmed, screening for and then treating autonomic dysfunction could represent an interesting potential approach to treating PSC fatigue which would itself warrant future exploration.

## Supporting information


**Fig. S1.** (a) Fatigue severity and (b) Significant fatigue frequency in the subgroups of PSC patients without (*n* = 16) and with associated IBD (*n* = 24) in comparison with community (*n* = 40) and IBD alone controls (*n* = 20). (c) Autonomic dysfunction symptoms in the subgroups of PSC patients with and without IBD and (d) the association between fatigue severity and autonomic symptom severity in the two groups. Open circles and broken line PSC only, solid circles and solid line PSC + IBD.

**Fig. S2.** (a) Fatigue severity and (b) Autonomic symptom severity in PSC and IBD patients with (*n* = 8) and without (*n* = 16) significant surgery (colectomy & ileostomy/pouch formation). (c) Fatigue severity in PSC patients treated (*n* = 25) and not‐treated (*n* = 15) with UDCA. (d) Fatigue severity in PSC patients compared to the Newcastle cohort of transplanted PSC patients (*n* = 11).
