# Supplementary figures and images for: Fatigue in primary sclerosing cholangitis is associated with sympathetic over‐activity and increased cardiac output
Source: Liver Int. 2014 Dec 4;35(5):1633–41. doi: 10.1111/liv.12709 (PMC4737110; doi:10.1111/liv.12709)

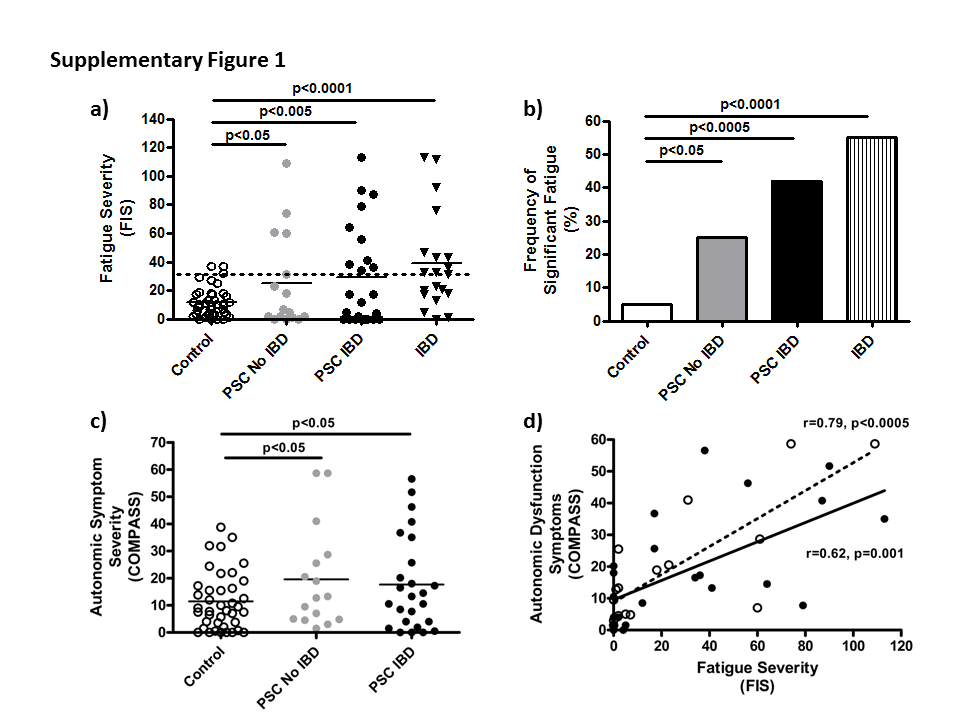

Supplement: Supplementary file 1 — Fig. S1. (a) Fatigue severity and (b) Significant fatigue frequency in the subgroups of PSC patients without (n = 16) and with associated IBD (n = 24) in comparison with community (n = 40) and IBD alone controls (n = 20). (c) Autonomic dysfunction symptoms in the subgroups of PSC patients with and without IBD and (d) the association between fatigue severity and autonomic symptom severity in the two groups. Open circles and broken line PSC only, solid circles and solid line PSC + IBD. [file LIV-35-1633-s001.tif]

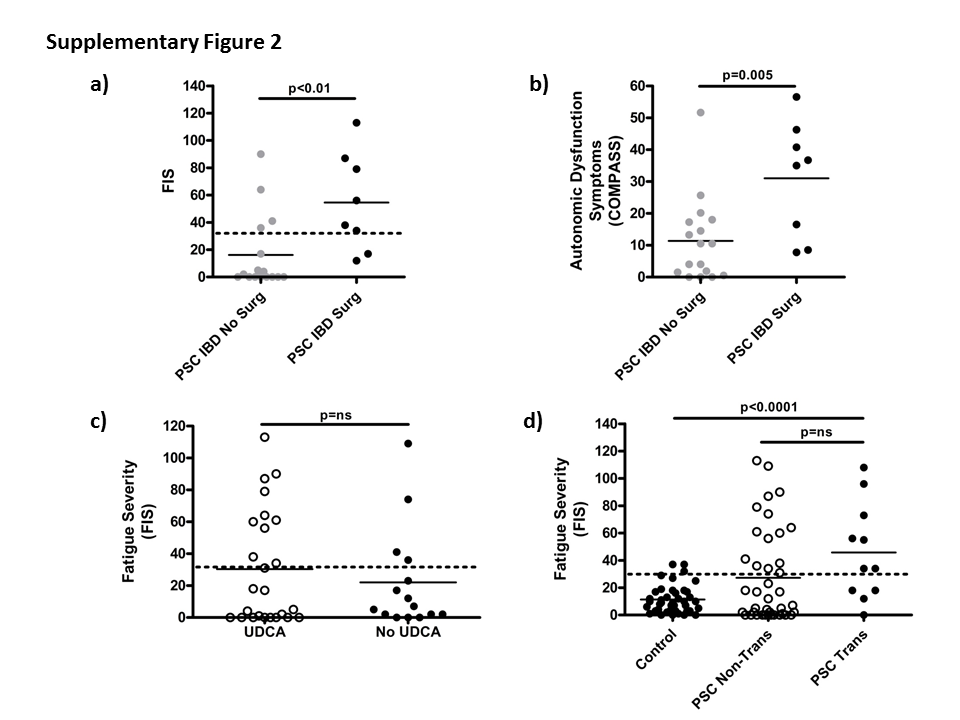

Supplement: Supplementary file 2 — Fig. S2. (a) Fatigue severity and (b) Autonomic symptom severity in PSC and IBD patients with (n = 8) and without (n = 16) significant surgery (colectomy & ileostomy/pouch formation). (c) Fatigue severity in PSC patients treated (n = 25) and not‐treated (n = 15) with UDCA. (d) Fatigue severity in PSC patients compared to the Newcastle cohort of transplanted PSC patients (n = 11). [file LIV-35-1633-s002.tif]
